# The direct disease burden of COVID-19 in Belgium in 2020 and 2021

**DOI:** 10.1186/s12889-023-16572-0

**Published:** 2023-09-04

**Authors:** Brecht Devleesschauwer, Lander Willem, Jure Jurčević, Pierre Smith, Aline Scohy, Grant M. A. Wyper, Sara Monteiro Pires, Nina Van Goethem, Philippe Beutels, Nicolas Franco, Steven Abrams, Dieter Van Cauteren, Niko Speybroeck, Niel Hens, Robby De Pauw

**Affiliations:** 1 Department of Epidemiology and Public Health, Sciensano, Brussels, Belgium; 2 Department of Translational Physiology, Infectiology and Public Health, Faculty of Veterinary Medicine, Ghent University, Merelbeke, Belgium; 3 Department of Family Medicine and Public Health, University of Antwerp, Antwerp, Belgium; 4 Centre for Health Economic Research and Modelling Infectious Diseases (CHERMID), Vaccine & Infectious Disease Institute, University of Antwerp, Antwerp, Belgium; 5 Institute of Health and Society (IRSS), Université Catholique de Louvain, Brussels, Belgium; 6 School of Health & Wellbeing, University of Glasgow, Glasgow, UK; 7 National Food Institute, Technical University of Denmark, Lyngby, Denmark; 8 Data Science Institute, Interuniversity Institute of Biostatistics and statistical Bioinformatics (I-BioStat), UHasselt, Hasselt, Belgium; 9 Namur Institute for Complex Systems (naXys) and Department of Mathematics, University of Namur, Namur, Belgium; 10 Institute of Health and Society, Université catholique de Louvain, Brussels, Belgium; 11 Department of Rehabilitation Sciences, Ghent University, Ghent, Belgium

**Keywords:** Burden of disease, COVID-19, Disability-adjusted life years, Years of life lost, Years lived with disability

## Abstract

**Background:**

Burden of disease estimates have become important population health metrics over the past decade to measure losses in health. In Belgium, the disease burden caused by COVID-19 has not yet been estimated, although COVID-19 has emerged as one of the most important diseases. Therefore, the current study aims to estimate the direct COVID-19 burden in Belgium, observed despite policy interventions, during 2020 and 2021, and compare it to the burden from other causes.

**Methods:**

Disability-adjusted life years (DALYs) are the sum of Years Lived with Disability (YLDs) and Years of Life Lost (YLLs) due to disease. DALYs allow comparing the burden of disease between countries, diseases, and over time. We used the European Burden of Disease Network consensus disease model for COVID-19 to estimate DALYs related to COVID-19. Estimates of person-years for (a) acute non-fatal disease states were calculated from a compartmental model, using Belgian seroprevalence, social contact, hospital, and intensive care admission data, (b) deaths were sourced from the national COVID-19 mortality surveillance, and (c) chronic post-acute disease states were derived from a Belgian cohort study.

**Results:**

In 2020, the total number of COVID-19 related DALYs was estimated at 253,577 [252,541 − 254,739], which is higher than in 2021, when it was 139,281 [136,704 − 142,306]. The observed COVID-19 burden was largely borne by the elderly, and over 90% of the burden was attributable to premature mortality (i.e., YLLs). In younger people, morbidity (i.e., YLD) contributed relatively more to the DALYs, especially in 2021, when vaccination was rolled out. Morbidity was mainly attributable to long-lasting post-acute symptoms.

**Conclusion:**

COVID-19 had a substantial impact on population health in Belgium, especially in 2020, when COVID-19 would have been the main cause of disease burden if all other causes had maintained their 2019 level.

**Supplementary Information:**

The online version contains supplementary material available at 10.1186/s12889-023-16572-0.

## Introduction

On the 11th of March 2020, the World Health Organization (WHO) declared the severe acute respiratory syndrome coronavirus 2 (SARS-CoV-2) outbreak as a pandemic [1]. In Belgium, the first infections with SARS-CoV-2 were reported by the end of February 2020. A rapid increase in cases resulted in a lockdown of the country on the 18th of March 2020 and the peak of the first wave was reached at the beginning of April 2020 [2]. After a national lockdown, virus circulation increased again, causing a high number of infections, hospitalizations, and casualties over different waves in 2020 and 2021 [3]. To prevent the further transmission of SARS-CoV-2 in 2020 and 2021, people were encouraged to maintain protective public health measures such as physical distancing, extensive testing and tracing followed by isolation of cases and quarantine of close contacts, wearing facemasks in public, and from 2021 onwards when vaccines became available, COVID-19 vaccination.

Monitoring population health is essential to identify where health improvements are required, to develop targeted prevention and intervention strategies, and to guide policies for addressing unmet health needs and identifying health priorities. To this end, burden of disease assessments allow estimates of the occurrence of morbidity and mortality to be translated into a single measure, the Disability-Adjusted Life Years (DALYs), thereby facilitating comparisons with other causes of disease and injury [4]. This composite DALY measure is calculated by standardizing the effects of morbidity and mortality as health loss over time [5]. For the morbidity component, severity distributions and disability weights (DWs) indicate how burdensome living with a disease is; whereas age-conditional life expectancies reveal the disease burden caused by premature mortality, implying that death at a younger age constitutes a greater burden, ceteris paribus [6]. The direct impact of COVID-19 on individual health occurs via three main pathways, (1) the acute infectious phase with a morbidity component (e.g., feeling sick, being hospitalized, etc.), (2) the acute infectious phase with a mortality component (i.e., COVID-19 related fatalities), and (3) a chronic phase with long-lasting morbidity beyond the period of the acute symptomatic infection. During the acute symptomatic stage of the infection, COVID-19 can cause a wide spectrum of symptoms, such as nausea, coughing, and loss of taste and smell that could reduce the quality of life (expressed by DWs between 0 and 1, with 0 reflecting “perfect health” and 1 reflecting “equivalent to death”), and, in the most extreme case, results in death [7]. If symptoms persist beyond the acute infectious period, i.e., post-COVID-19 condition, symptoms could contribute to a chronic morbidity component, whereby some symptoms persist for longer periods [8]. Additionally, there is mounting evidence that COVID-19 leads to long-term elevated risks of relatively common causes of disease burden, such as cardiovascular and neurological complications, including ischemic and hemorrhagic stroke, cognition and memory disorders, peripheral nervous system disorders, episodic disorders, extrapyramidal and movement disorders, mental health disorders, musculoskeletal disorders, sensory disorders, Guillain–Barré syndrome, and encephalitis or encephalopathy [9, 10]. However, quantifying the indirect disease burden resulting from COVID-19 is a challenge due to its complex and multi-faceted nature. The estimation of DALYs requires the existence of and accessibility to a wide variety of data sources to match the different disease states of the underlying YLLs and YLDs model (e.g., cases, hospitalizations, and deaths).

Clearly, pandemics, such as COVID-19, also exert indirect impacts beyond COVID-19 patients and their personal family, friends and employers [11]. For example, perceived risks of acquiring COVID-19 and imposed non-pharmaceutical interventions influenced the frequency and severity of mental health issues [12]. Furthermore, both risk perceptions and capacity problems due to COVID-19, caused postponements and cancellations of unrelated preventive and curative healthcare [13–15]. Even if these indirect impacts are beyond the scope of the current study, it represents an essential first step, by attempting to estimate the extent of the direct impact on population health in Belgium, while focusing on the first two calendar years of the pandemic (2020 and 2021).

This study aimed to estimate the COVID-19 burden including direct acute and direct post-acute consequences using the DALY approach at the national level in Belgium anno 2020 and 2021 by relying on a consensus burden of disease model, developed for COVID-19 by the European Burden of Disease Network [16].

## Methods

The current study has adopted the European Burden of Disease Network consensus disease model for COVID-19 using a prevalence-based approach to estimate COVID-19 related DALYs [16]. DALYs reflect the healthy life years lost due to diseases and constitute a morbidity component, i.e., Years Lived with Disability (YLDs), and a mortality component, i.e., Years of Life Lost (YLLs). Estimates of the burden of disease were calculated at the Belgian national level. Direct inputs for the calculation of the disease burden were (1) the output from an age-structured stochastic compartmental model, which has been calibrated on age-specific seroprevalence data, confirmed COVID-19 hospitalizations and deaths among others, while accounting for changing contact patterns, vaccination uptake and different SARS-CoV-2 variants of concern [17], (2) a cohort in the Belgian population called COVIMPACT including 6,913 participants who reported to experience COVID-19 symptoms after a positive test, for which at least two follow-up points were available (December 2022) to assess the proportion of acute COVID-19 patients that develop post-acute long-lasting symptoms [18], and (3) COVID-19 mortality surveillance systems in Belgium. The total disease burden in DALYs is calculated as the sum of the morbidity in YLDs and mortality in YLLs components.

### Morbidity (Years Lived with Disability)

#### Data sources

YLDs were estimated using the person-time estimates from the stochastic compartmental model developed for Belgium. This stochastic transmission model as described by Abrams et al. (2021) has previously been adapted to include vaccination and the emergence of several variants of concern. [17] The model is calibrated on early seroprevalence data, genomic surveillance data, hospital admission data, mortality data, and social contact data from the Belgian CoMix survey [19]. Gradually accumulating and waning of naturally-acquired immunity in the population is accounted for, as well as immunity induced by vaccination. The vaccine-type and age-specific uptake over time is based on the reported data by Sciensano, derived from Epistat. The model results contain stochastic variation in the transmission process and parameter uncertainty based on 40 model parameter configurations. The calibration process relies on likelihood-based MCMC sampling until convergence of a single MCMC chain after which 40 posterior samples of the joint parameter distribution are generated. Each of these posterior samples is then used to simulate 10 stochastic realizations of the model output, leading to a total of 400 simulated values for, for example, the total number of (a)symptomatic infections per day between the 1st of March 2020 (the beginning of the COVID-19 crisis) and the 31st of December 2021 stratified by 10-year age groups. Symptomatic infections were characterized in three compartments: (1) not requiring hospitalization; (2) requiring hospitalization but not intensive care unit admission; and, (3) requiring intensive care unit admission. Based on these estimates, we calculated the total number of person-years by compartment (i.e., health state), whereby the number of people that were in each of the aforementioned compartments and the time spent in the compartment (i.e., duration of illness) was considered. As the proportion of people that continue to experience symptoms after the acute symptomatic phase is currently unknown and debated [8, 20, 21], we have estimated the proportion of patients that develop post-acute consequences and the duration of these post-acute consequences based on the longitudinal Belgian COVIMPACT cohort study, which, as described by Smith et al. (2022), was designed for that purpose [18, 22]. This population-based cohort included a total of 6,913 participants in December 2022 with an age between 18 and 102 years. 35.1% of participants reported to be men, 64.8% to be women, and 0.1% described themselves as the category other. The COVIMPACT study assesses self-reported symptoms at the time of infection, and at regular 3-month intervals after infection between the 29th of April 2021 and the 31st of December 2022. In the calculations, YLDs caused by COVID-19 were only considered during the respective calendar years, i.e., the prevalence approach [23].

#### Health states

The different health states as defined by the European Burden of Disease Network consensus model [16] that were considered in this study are listed in Table 1. A total of five different health states are described, of which the health states “asymptomatic”, “mild/moderate”, “severe”, and “critical” were approximated by the output from the stochastic compartmental model for Belgium. The health state “post-acute consequences” was approximated based on the results from the COVIMPACT study, where information on “tiredness” and “pain” was used to align the study outcomes with the definition of the “post-acute consequences” health state. In total 180 of 5,866 participants (3.1%, 95% uncertainty interval: 2.6 − 3.6%) reported being “tired” and “feeling pain over the body”, as specified in the health state definition, three months after confirmation of the infection. The distribution of the duration of symptoms was inferred by considering different parametric distributions for which the parameters were estimated while accounting for left-truncation and the interval-censored nature of the data. More specifically, a truncated log-normal distribution with mean and standard deviation parameters µ and σ equal to 5.10 (95% CI: 5.07–5.13) and 0.26 (95% CI: 0.24–0.27), respectively, was selected. Considering a minimum duration of 3 months (90 days), the average duration of post-acute symptoms in patients that developed post-acute consequences within this cohort equalled 260 days with a standard deviation of 44.9 days. More detailed information on the applied methodology to model the “post-acute consequences” health state can be found in Appendix 1. The total number of person-years in this health state was calculated by applying a microsimulation decision tree model with 400 iterations, whereby each case that would transit from a “symptomatic” compartment (i.e., community-based cases, cases requiring hospitalization, and cases requiring intensive care unit admission) to the “recovered” compartment in the compartmental model had a probability of 3.1% (95% CI: 2.6 − 3.6%) to develop long-term symptoms of “tiredness” and “bodily pain” with a duration generated from the aforementioned truncated log-normal distribution.

**Table 1:** Health states and disability weights for estimating the COVID-19 burden in Belgium

**Health state**	**Description**	**Data input proxy**	**Disability weight (95% uncertainty interval)**
Asymptomatic	Has infection but no symptoms	Estimates of suspected asymptomatic community cases	0 (0.000 – 0.000)
Mild/Moderate	Has fever and aches, and feels weak, which causes some difficulty with daily activities.	Positive (and/or suspected) cases community cases	0.051 (0.032 – 0.074)
Severe	Has high fever and pain, and feels very weak, which causes great difficulty with daily activities	Positive (and/or suspected) cases requiring a non-intensive care hospitalization	0.133 (0.088-0.190)
Critical	Intensive care unit Admission	Positive (and/or suspected) cases requiring intensive care hospitalization	0.655 (0.579-0.727)
Post-acute consequences	Is always tired and easily upset. The person feels pain all over the body and is depressed		0.219 (0.148-0.308)

The uncertainty around the disability weights was estimated through a Monte Carlo simulation approach as described in Salomon et al. (2015) and Haagsma et al. (2015) [24, 25]. These DWs are defined per day lived in each health state, as they are multiplied with the estimated number of person-days per year in each health state to arrive at YLD

#### Disability weights

DWs vary between 0 (no impact, perfect health) to 1 (equivalent to death) and reflect how incapacitating a health state is for a panel. For the different health states considered in this study, DWs were based on the Global Burden of Disease (GBD) 2019 study for infectious diseases (i.e., lower respiratory tract infections) [24]. For critical care, the DW was based on the European Disability Weights Study (Table 1) [25].

For each age category, YLDs were calculated by summing across all health states *i*, the product of the number of person-years (*N*_*i*_), and the corresponding disability weight (*DW*_*i*_) [16]:

$$YLDs=\sum _{i}{N}_{i}{DW}_{i}$$

The total YLDs are calculated by summing age-specific YLDs.

### Mortality (Years of Life Lost)

YLLs were calculated using an extracted dataset (extracted in December 2022) of the epidemiological mortality surveillance of COVID-19 in Belgium [26, 27]. This database is managed by Sciensano, the Belgian institute for health. Sciensano adopted an inclusive definition for COVID-19 death notifications, by also including possible COVID-19 related deaths, whereby patients met the following criteria: “at least one of the following symptoms that appear with no other obvious cause: cough, dyspnoea, thoracic pain, acute anosmia or dysgeusia” or “at least two of the following symptoms with no other obvious cause: fever, muscle pain, fatigue, rhinitis, sore throat, headache, anorexia, watery diarrhoea, acute confusion, sudden fall” or “deterioration if the patient shows chronic respiratory symptoms”. This resulted in a robust correlation between COVID-19 and all-cause mortality [2, 26]. A death was classified as a COVID-19 related death if it occurred in “a confirmed, radiologically-confirmed or possible case that occurred in any setting (hospitals, at home, nursing homes, and other settings) unless a clear alternative cause of death unrelated to COVID-19 is identified”. The database includes all COVID-related deaths during the first two calendar years of the pandemic (2020–2021). More information on the COVID-19-related mortality has been published elsewhere (see Renard et al., 2021 [26]). Missing data on age and sex were imputed by its marginal or conditional probability as described in Appendix 2.

 For each age group, YLLs were estimated by multiplying the number of deaths due to COVID-19, denoted by D, by the specific remaining life expectancy *RLE* from the GBD reference life table 2019 [28]:

$$YLLs=D\times RLE$$

The total YLLs because of COVID-19 was obtained by summing all age-specific YLL estimates. The calculated YLLs in each respective calendar year considered all expected years of life lost (also beyond the respective calendar year) [23]. Future life years were not discounted in this analysis.

### Burden of disease (DALYs)

The total disease burden of COVID-19 was estimated in absolute DALYs, in DALYs per 100,000 inhabitants, and in age-standardized DALYs per 100,000 individuals using the Belgian age distribution obtained from the Belgian statistical office (Statbel), which reflects the Belgian population on the 1^st^of January 2020 and 2021 [29]. After calculating the DALYs, we compared the estimates for 2020 and 2021 with the latest GBD DALY results for the top ten health conditions ranked at GBD level 3 for Belgium in 2019. We applied no discounting on the DALYs, YLLs, or YLDs components in the estimation.

### Model uncertainty

For the calculations of the YLLs we relied on the Belgian COVID-19 mortality register, whereby we did not redistribute causes of deaths. To account for the uncertainty in the remaining life expectancy tables, we based the YLLs calculation on the GBD 2019 life table, and performed a sensitivity analysis by calculating the YLLs based on the Belgian national life table. However, the GBD 2019 life table and Belgian national lifetable refer to different constructs. More specifically, the GBD life table was constructed based on the lowest observed age-specific mortality rates by location and sex across all estimation years from all locations with populations over 5 million in 2016. The Belgian national life tables on the other hand indicate how a generation would die out throughout its existence, if it was exposed to the same mortality conditions per age as in the specified year. The results of this sensitivity analysis can be found in Appendix 3. For the YLDs, the model applied a Monte Carlo approach including 1000 replications to account for the uncertainty with respect to (1) the disease model (i.e., the DWs in the different health states as described in Appendix 4), (2) the estimates of the health-state specific person-years (i.e., based on the stochastic compartmental model) [30], (3) the estimated probability of having long-term symptoms, and (4) the estimated distribution of the duration of long-term symptoms. In the first step, the 400 iterations from the stochastic compartmental model and the 400 iterations from the microsimulation model for long-COVID symptoms were resampled to obtain 1000 iterations. In a second step, the uncertainty of the different disability weights was approximated by a Beta distribution that matches the mean and 95% uncertainty interval (betaExpert-function as available from the “prevalence” package in R [31, 32]) yielding a random sample of 1000 DW estimates per health state. In a third step, the uncertainty for post-acute symptoms was modelled considering the uncertainty around the proportion of patients that would develop post-acute symptoms and their duration. To approximate the proportion of patients that could develop post-acute COVID-19 related symptoms, the same Beta distribution approach as described earlier was applied. Uncertainty related to the duration of post-acute symptoms, which was described by a truncated log-normal distribution, was accounted for by (1) sampling from a normal distribution with mean equal to the µ and σ parameter estimates and standard deviation the standard error around these estimates to account for the uncertainty around the parameter estimates of the log-normal distribution, and by (2) sampling a random duration based on a log-normal distribution characterized by the sampled parameters in step (1). Uncertainty intervals were defined as the 2.5th and 97.5th percentile of the specific disease estimated distributions.

## Results

In Belgium, the total observed person-time of COVID-19 cases in all health states was estimated at 27,499 person-years (95% uncertainty interval: [24,740 − 30,086]) in 2020, of which hospitalizations in intensive care accounted for 362 person-years [338–385], community-based cases with severe symptomatology accounted for 1,684 person-years [1,598-1,765], community-based cases with mild/moderate symptomatology accounted for 12,880 person-years [10,445 − 14,909], and post-acute symptoms accounted for 12,574 person-years [11,101 − 14,002]. In 2021, the observed person-time of all COVID cases was estimated at 49,732 person-years [45,032–54,911], of which hospitalizations in intensive care accounted for 432 person-years [407–457], community-based cases with severe symptomatology accounted for 1,610 person-years [1,531-1,695], community-based cases with moderate symptomatology accounted for 15,620 person-years [12,815 − 18,452], and post-acute symptoms accounted for 32,070 person-years [28,106 − 36,030]. The number of reported COVID-related deaths was 19,846 in 2020, and 8,557 in 2021. A visual representation of the time spent in each health state by age and time can be found in Appendix 5 A and 5B.

**Fig. 1:**
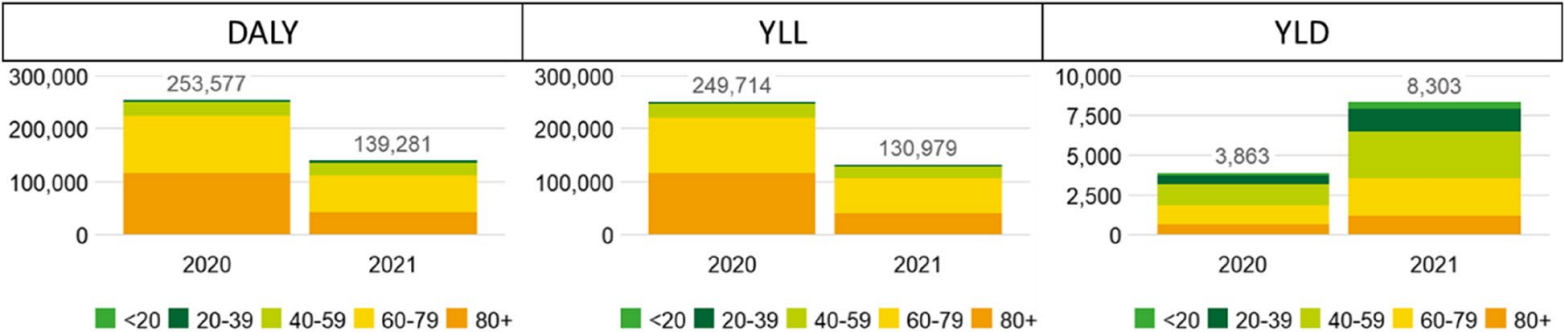
Total estimated disease burden of COVID-19 expressed as Disability-Adjusted Life Years (DALYs) per age category for the period 2020–2021. YLLs = Years of Life Lost; YLDs = Years Lived with Disability

As shown in Table 2 and depicted in Fig. 1, COVID-19 has led to 253,577 DALYs [252,541 − 254,739] in 2020 and 139,281 DALYs [136,704 − 142,306] in 2021. Most of the disease burden was associated with premature mortality (proportion of YLLs equal to 98.5% in 2020 and 94.0% in 2021). The disease burden was unequally distributed among age categories with 45.9% and 29.6% of the total disease burden occurring in the oldest (80+) age category in 2020 and 2021, respectively, and 41.9% and 49.7% in people with an age between 60 and 79 years in 2020 and 2021, respectively. The total disease burden was the lowest in the population less than 20 years old (0.3% and 0.4% in 2020 and 2021, respectively).

**Table 2:** Estimated disease burden (mean and 95% uncertainty interval) of COVID-19 in Disability-Adjusted Life Years (DALYs), Years Lived with Disability (YLDs) and Years of Life Lost (YLLs) by age category and total for the calendar years 2020 and 2021

	**2020**			**2021**		
	YLDs	YLLs	DALYs	YLDs	YLLs	DALYs
**Absolute**						
	158 [111-209]	523	681 [634-731]	422 [289-588]	161	583 [450-749]
20-39 years	571 [403-759]	3,205	3,776 [3,609-3,964]	1,435 [988-1,961]	2,896	4,332 [3,884-4,858]
40-59 years	1,315 [940-1,728]	25,111	26,426 [26,051-26,839]	2,874 [1,977-3,872]	21,01	23,884 [22,987-24,882]
60-79 years	1,176 [882-1,517]	105,183	106,359 [106,065-106,700]	2,416 [1,666-3,322]	66,813	69,229 [68,480-70,135]
80+ years	643 [490-812]	115,693	116,336 [116,182-116,505]	1,156 [805-1,583]	40,098	41,254 [40,903-41,681]
***Total***	**3,863 [2,826-5,025]**	**249,714**	**253,577 [252,541-254,739]**	**8,303 [5,726-11,327]**	**130,979**	**139,281 [136,704-142,306]**
**Rate per 100,000**						
	6.2 [4.3-8.1]	40.3	46.5 [44.7-48.5]	16.4 [11.3-22.9]	12.6	29.0 [23.9-35.5]
20-39 years	19.7 [13.9-26.2]	217.5	237.2 [231.4-243.7]	49.5 [34.1-67.6]	196.2	245.7 [230.2-263.8]
40-59 years	42.5 [30.4-55.8]	1,601.3	1,643.8 [1,631.7-1,657.2]	92.9 [64.0-125.2]	1,345.3	1,438.3 [1,409.3-1,470.6]
60-79 years	51.8 [38.8-66.8]	10,019.4	10,071.2 [10,058.3-10,086.2]	104.3 [71.9-143.4]	6,088.2	6,192.5 [6,160.2-6,231.7]
80+ years	97.9 [74.5-123.7]	40,305.3	40,403.2 [40,379.8-40,429.0]	178.5 [124.3-244.5]	12,678.6	12,857.0 [12,802.9-12,923.0]
***Total***	***33***.***6 [24***.***6-43***.***7]***	***2,172.8***	***2,206.4 [2,197.4-2,216.5]***	***72.1 [49.7-98.3]***	***1,136.8***	***1,208.9 [1,186.5-1,235.2]***
***Standardized***^***a***^				***72.0 [49.7-98.2]***	***1,132.4***	***1,204.4 [1,182.1-1,230.7]***

^a^Standardized according to the population in Belgium on the 1^st^ of January 2020 based on groups with 20-year age bands

### Morbidity (years lived with disability)

In 2020, a total of 3,863 YLDs [2,826-5,025] were observed due to acute symptomatic COVID-19 infections and post-acute symptoms after infection including mild/moderate cases (654 YLDs; 16.9%), severe cases (223 YLDs; 5.8%), critical cases (236 YLDs; 6.1%), and post-acute cases (2,750 YLDs; 71.2%). In 2021, a total of 8,303 YLDs [5,726 − 11,327] were estimated due to acute symptomatic COVID-19 infections including mild/moderate cases (791 YLDs; 9.5%), severe cases (213 YLDs; 2.6%), critical cases (213 YLDs; 3.4%), and post-acute cases (7,016 YLDs; 84.5%). The percentage of observed DALYs attributable to morbidity is highest among people younger than 20, whereby it was higher in 2021 (72.4%) as compared to 2020 (23.3%). In the group aged between 20 and 39, 15.1% of the disease burden was attributable to YLDs in 2020 and 33.1% in 2021. In the older age groups, YLDs accounted for less than 15.0% of the total disease burden in 2020 and 2021.

### Mortality (years of Life Lost)

During the first year of the pandemic, 249,714 years of life were lost due to premature mortality in Belgium. In 2021, there was a significant decrease in premature mortality, with an estimated 130,979 YLLs. The YLLs per death was estimated at 12.6 in 2020 compared to 15.3 in 2021. The percentage of DALYs attributable to mortality was the highest among people aged between 60 and 79 years, and older than 80 years. In 2020, it was estimated at 98.9% and 99.4% in the age groups of 60 and 79 years, and older than 80 years, respectively. In 2021 it was estimated at 96.5% and 97.2% in the age groups of 60 and 79 years, and older than 80 years, respectively. Over 80.0% of the YLLs occurred in the group of people with an age over 60 years in 2020 and 2021. YLLs estimates based on the Belgian national life expectancy table in comparison with the GBD 2019 table can be found in Appendix 3.

## Discussion

This study aimed to estimate the direct COVID-19 disease burden in Belgium during the first two calendar years of the pandemic. We estimated a total COVID-19 disease burden of 253,577 DALYs [252,541 − 254,739] in 2020, and a total of 139,281 DALYs [136,704 − 142,306] in 2021. More than 80% of the observed disease burden occurred in the population of 60 years of age and older. Of the total observed disease burden, more than 90% could be attributed to premature mortality.

Compared to the disease burden estimates for 2019 by the GBD, the disease burden of COVID-19 in 2020 was 40,254 DALYs higher than ischemic heart disease, the disease with the largest contribution to the overall disease burden, and 183,277 DALYs more than lower respiratory infections [33]. Note that COVID-19 only contributed to the disease burden in the final 10 months of the year 2020, whereas the estimates for these two other causes covered the full year in 2019. If disease burden estimates for all other causes in 2019 could be transferred to 2020, COVID-19 would be the most important cause of disease burden in 2020. The COVID-19 disease burden substantially dropped from 253,577 DALYs in 2020 to 139,281 DALYs in 2021, and COVID-19 would then rank as the 6th most important cause of illness in 2021, in between chronic obstructive pulmonary disorder (142,937 DALYs) and stroke (134,777 DALYs) [33]. In addition, COVID-19 would supersede the impact of lower respiratory infections, which is the next group of vaccine-preventable diseases in the top 20, and ranked at place 13th in 2019. When limiting to the morbidity component (YLDs), the disease burden of COVID-19 in 2020 could be ranked as 57^th^ between gout and urticaria, while in 2021, the observed disease burden of COVID-19 could be ranked as 44^th^ between other sense organ diseases and viral skin diseases. These comparisons should however be interpreted with caution, as it is so far not yet clear how the COVID crisis affected the disease burden of non-COVID conditions.

Our results show that, although public health measures such as telework, mask-wearing, isolation, test strategies, social distancing, and travel restrictions were adopted in Belgium to limit the impact of COVID-19 on population health (see Appendix 6 for more details), the estimated burden in 2020 remained high [34]. Although our study does not allow to infer causality for the large drop in DALYs in 2021 compared to 2020, the biomedical plausibility that the vaccination campaign, which started in Belgium on the 28^th^ of December 2020, caused the change in remaining disease burden is high. Indeed, 2021 saw increasing vaccine uptake, reduced social distancing and other public health measures, more severe SARS-CoV-2 variants and a shift in burden towards less lethal, less severe and more prevalent COVID-19 cases, as evidenced by the randomised controlled vaccine trials [35]. In addition, the build-up of natural immunity against SARS-CoV-2 could also play an important role in the observed drop. Even though social distancing and other public health measures were reduced in 2021 and more severe SARS-CoV-2 variants emerged, we observed a shift in burden towards less lethal, less severe, and more prevalent COVID-19 cases, as one would expect from the evidence provided by the randomized controlled vaccine trials. This could also partly explain the increased YLD in 2021 compared to 2020.

In Europe, DALY estimates have been calculated using the European Burden of Disease Network consensus disease model for COVID-19 during the first calendar year of the pandemic in Denmark, Ireland, Scotland, Germany, Malta, the Netherlands, and France [6, 36–41]. Caution is required when comparing burden of disease estimates across countries, as differences between countries in terms of COVID-19 disease burden are partly explained by differences in data that is available in those countries. Although some countries applied models that account for possible over- or underreporting of symptomatic cases or deaths, the data that is sourced in the consensus disease model remains vulnerable to the country-specific situation. For example, using a broad definition for COVID-19 related death or differences in testing strategy in the first months of the pandemic will affect the disease burden estimates. The estimated COVID-19 burden in Belgium in 2020 (2,206.4; 95% uncertainty interval: 2,197.4-2,216.5) was higher compared to the reported burden in other European countries in 2020, our estimated total burden per 100,000 inhabitants was 1.18 times higher than the burden estimated for Scotland [37], 1.35 times higher compared to the Netherlands [40], 2.03 times higher than Malta [42], and 1.50 times higher than France [41]. Other countries (i.e., Ireland, Germany, and Denmark) either used a different standardized life table or did not consider post-acute symptoms. Although the share of the disease burden attributable to morbidity is rather small, the study for Scotland also applied an SEIR compartmental model to estimate the number of community-based cases, which might partly explain the relatively higher burden estimates for Scotland and Belgium during the first year of the pandemic. In 2020, the test and tracing strategy might not have been optimized, resulting in many missed reported cases during the first months of the pandemic. In addition, the death count in Belgium is based on a more inclusive definition for a COVID-19-related death compared to other European countries [2, 26, 27]. To date, no information on COVID-19 mortality is available based on death certificates as the compilation of causes of death from these certificates has a delay of 2 to 3 years. Once available, a validity check could be performed against these certificates.

Although the incidence of observed COVID-19 infections remained high in 2021, the overall age-standardized disease burden decreased from 2,206.4 [2,197.4-2,216.5] per 100,000 inhabitants in 2020 to 1,204.4 [1,182.1-1,230.7] per 100,000 inhabitants. This drop is mainly attributable to changes in mortality as only a relatively small share (< 10.0%) of the total burden of COVID-19 was attributable to morbidity. Although the share of morbidity is lower compared to mortality in the estimated DALYs, there is a danger of over-interpreting YLDs health estimates in isolation. For example, preventing cases with severe symptomatology would probably not only decrease the YLDs related to severe symptomatology but also prevent YLLs related to severe symptomatology, as patients with severe symptoms were more likely to decease. The share of the disease burden attributable to morbidity was substantially larger in the younger group. Studies have shown that there is a strong age-mortality relation in the population of patients diagnosed with COVID-19, with a higher risk to decease in older ages [43]. However, the group of adolescents and young adults are more vulnerable in terms of mental health after an acute COVID-19 infection compared to, for example, older individuals [44, 45]. However, this was not accounted for in this study. The total disease burden of COVID-19 attributable to its post-acute consequences has increased from 71.2% to 2020 to 84.5% in 2021, a finding similar to other European reports. Scotland, for example, reported the highest contribution of post-acute consequences to YLDs (76%) [37], followed by Malta (60%) [42].

### Strengths and limitations

This is the first study that estimated the disease burden of COVID-19 in Belgium up to 2021. We successfully combined national data sources and models on COVID-19 infections, hospitalisations, seroprevalence, variants of concern, vaccination and mortality to capture the direct impact of COVID-19 on population health. Uncertainties around the disease model and the person-year estimates were considered, and we have highlighted their impact on YLDs and DALYs estimates. To estimate the YLLs, the standard GBD life table was used to facilitate the comparison of these results with other studies, achieve consistency with other cause-specific disease burden estimates. However, we acknowledge the ongoing disagreements on the use of standard life table [46]. Therefore, we have performed a sensitivity analysis (which can be found in Appendix 3) by comparing the estimated YLLs based on the GBD with the estimated YLLs based on the Belgian national life table. Lastly, we adopted a standardized approach based on a consensus model that allowed us to produce comparable estimates with other countries.

Besides its strengths, the current study had some limitations. First, the current Belgian compartmental model made, for example, no distinction between men and women, which made between-sex comparisons of the burden of disease impossible. The stochastic model is calibrated on hospitalizations, which is not prone to testing strategies, but assumes a direct link between hospitalizations and cases. Next, the YLLs were estimated based on an ad hoc COVID-19 mortality surveillance set up during a crisis situation including a broad definition of COVID-19, and not using the standard practice of ICD-10 nomenclature. These results may therefore over- or underestimate the YLLs, however, until causes of death estimates are available, this assessment cannot be made. Nevertheless, this approach allowed to estimate the COVID-19 mortality during the pandemic based on the latest available data. Third, YLDs of post-acute consequences of COVID-19 were calculated using transition probabilities based on a new cohort with only a limited time of follow-up. The estimated cases of post-acute symptoms of COVID-19 should be interpreted with caution, as there is an ongoing debate on the definition of post-acute consequences of COVID-19 [47, 48], and if these post-acute consequences are causally related to the acute infection. These discussions contribute to the widely varying estimates that have been reported so far [49]. In the methodology to estimate the disease progression, we fitted a parametric survival model with a log-normal distribution, which is only an approximation of the duration in symptoms. As these underlying assumptions might not hold, their corresponding estimates should be interpreted with caution. Lastly, we do not account for comorbidities but we estimate the disease burden of COVID-19 by age, which we assume as proxy for the frailty of patients (e.g., by suffering from comorbidities).

### Implications for public health

Our estimates of DALYs due to COVID-19 in 2020 and 2021, and its YLLs and YLDs composites, highlight the direct and immediate impact in terms of mortality and morbidity in acute infections and post-acute consequences of COVID-19 among the Belgian population. This study highlights the importance of available data collection practices especially in the context of more specific information on symptomatology, symptom duration, and post-acute consequences of COVID-19. These estimates are useful for informing public health decision-makers regarding the COVID-19 burden among subgroups of the population who were the most affected by mortality and to target the high-risk sub-groups to limit the impact of COVID-19 on mortality estimates. The current study identified that COVID-19 likely caused more disease burden during the first calendar year (2020) compared to the second year (2021), which emphasizes the importance of preparedness for potential future pandemics in an attempt to reduce the initial burden of a pandemic during its initial months, when there is no natural immunity against the virus, or no vaccine available. Furthermore, it is important to not only evaluate the direct impact of the COVID-19 pandemic on population health, but also study the long-term indirect impact such as the disruption of the utilisation of vital health care services [50], increased mental health problems [51], risk of increase in health inequalities [52], and economic losses. Therefore, future studies should include measures at regional levels, and attempt to measure the indirect impact of COVID-19 as a risk factor on the collateral damage to health care services, mental health and increase of incidences of non-communicable diseases associated with COVID-19.

## Conclusion

COVID-19 had an important effect on the population health in Belgium during its first two calendar years. Most of the direct population health loss was attributable to mortality, and mainly carried by people aged 60 and above. Our results highlight that the post-acute consequences of COVID-19 might strongly contribute to COVID-19 morbidity and can have a considerable impact on population health with, consequently, a higher burden on the health care system and health-related quality of life. Further research is required to evaluate the direct longer term and indirect impact of COVID-19 and study potential inequalities associated with the health burden of COVID-19.

### Supplementary Information

**Additional file 1. Appendix 1. **Modeling long-lasting symptoms after a confirmed SARS-CoV-2 infection based on the longitudinal follow-up data from the Belgian COVIMPACT study. **Appendix 2.** Imputation of the COVID-19 mortality dataset. **Appendix 3.** Comparison of YLL estimates of the COVID-19 burden in Belgium using the Global Burden of Disease Remaining Life Expectancy Table (2019) and the Belgian National Life Expectancy Table (2019). **Appendix 4.** Overview of distributions of disability weights approximated by a beta distribution using the 95% uncertainty interval around the DW. **Appendix 5A.** Figure with an overview of prevalent cases and deaths in each health state for the year 2020 by day and age category. **Appendix 5B.** Figure with an overview of prevalent cases and deaths in each health state for the year 2021 by day and age category. **Appendix 6.** Table with an overview of important public health measures taken in response to the COVID-19 crisis during the period 2020-2021.

## Data Availability

The datasets generated and/or analysed during the current study are available in the COVID-19 repository from Sciensano, https://epistat.sciensano.be/covid/.
