# Supplementary material for: The direct disease burden of COVID-19 in Belgium in 2020 and 2021
Source: BMC Public Health. 2023 Sep 4;23:1707. doi: 10.1186/s12889-023-16572-0 (PMC10476343; doi:10.1186/s12889-023-16572-0)
Supplement: Supplementary file 1 — Additional file 1. Appendix 1. Modeling long-lasting symptoms after a confirmed SARS-CoV-2 infection based on the longitudinal follow-up data from the Belgian COVIMPACT study. Appendix 2. Imputation of the COVID-19 mortality dataset. Appendix 3. Comparison of YLL estimates of the COVID-19 burden in Belgium using the Global Burden of Disease Remaining Life Expectancy Table (2019) and the Belgian National Life Expectancy Table (2019). Appendix 4. Overview of distributions of disability weights approximated by a beta distribution using the 95% uncertainty interval around the DW. Appendix 5A. Figure with an overview of prevalent cases and deaths in each health state for the year 2020 by day and age category. Appendix 5B. Figure with an overview of prevalent cases and deaths in each health state for the year 2021 by day and age category. Appendix 6. Table with an overview of important public health measures taken in response to the COVID-19 crisis during the period 2020-2021. [file 12889_2023_16572_MOESM1_ESM.docx]

**Appendix 1**: Modeling long-lasting symptoms after a confirmed SARS-CoV-2 infection based on the longitudinal follow-up data from the Belgian COVIMPACT study.

The COVIMPACT study assesses the evolution of patients that tested positive for a SARS-CoV-2 infection and the evolution of their symptomatology to evaluate possible long-term symptoms. To evaluate the burden of disease attributable to post-acute consequences, we have estimated the duration of symptoms related to the disease state as proposed by the European Burden of Disease Network consensus model [13]. The study includes 6,913 participants, for which at least two follow-up points are available. Study participants had an age between 18 and 102 years old with an average age of 44.4 years old. The study population included mainly women (64.8%), followed by men (35.1%), and only a small group reported to be in the category of others (0.1%). To calculate the number of person-years that are associated with long-COVID, a binomial model was applied to estimate the proportion of acute patients that suffer from long-COVID symptoms (i.e., symptoms for at least 90 days or 3 months), and a time-to-event model was applied to estimate the duration of long-COVID symptoms conditional on the development of long-COVID symptoms. As the assessment of participants in the COVIMPACT study only takes place at regular time intervals (i.e., around 3 months, 6 months, 9 months, and 12 months of follow-up), we have applied a parametric regression model for interval-censored data, which is available in the *R package icenReg*. Interval censoring occurs as the status of participants is known only up to an interval. If a participant declares symptoms related to post-acute consequences at *t_1_* and no longer at *t_2_*, all that is known is that the participants’ symptoms resolved in (*t_1_*, *t_2_*), rather than an exact time at which symptoms disappeared. In addition, we have corrected for truncation of the event time (defined as the time at which symptoms disappear), as we aim to estimate the duration of symptoms for patients that experience symptoms for more than 3 months. In the first phase, we assessed the fit of different parametric distributions, relying on maximum likelihood estimation, by visual inspection and the corresponding log-likelihood value reached for the ML estimates. As shown in **table 1**, the log-normal model had the best fit in terms of log-likelihood value (Table 1). A plot with the fitted distributions is depicted in **Figure 1**. Consequently, we have selected the log-normal distribution to estimate the duration of long-COVID symptoms. Afterward, we assessed the significance of sex and age as independent factors by including them in the model, whereby neither was significant at a 5% significance level.

***Table 1:*** *Table with log-likelihood estimates for the different distributions*

| **Model** | **Log-likelihood** |
| --- | --- |
| Weibull | -366 |
| Log-normal | -337 |
| Log-logistic | -350 |
| Exponential | -1123 |

***Figure 1:*** *Plot of the parametric fit according to a selection of distributions compared to the data in the COVIMPACT study.*


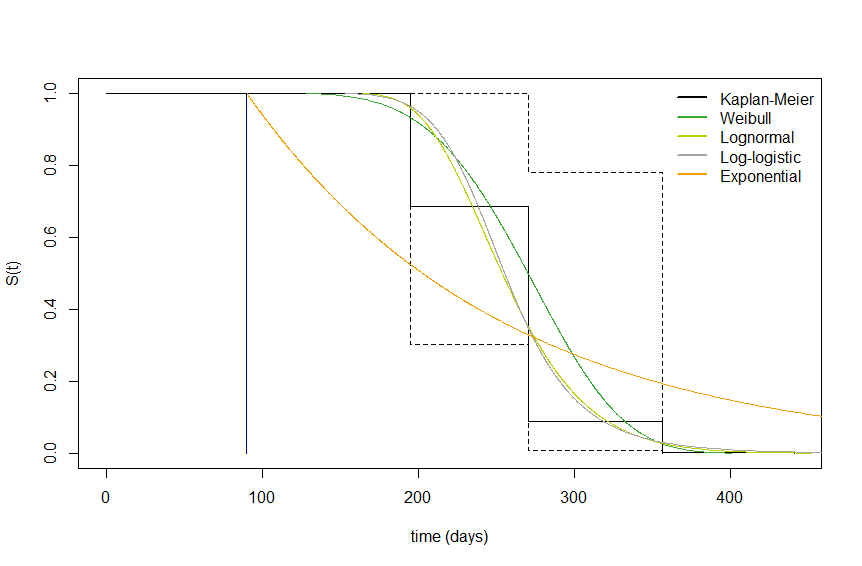


***Abbreviations****: S(t): Survival probability. The blue vertical lines depicts the entrance into the long-COVID cohort.*

**Appendix 2**: Imputation of the COVID-19 mortality dataset.

YLL and case fatality were calculated using an extracted dataset of the epidemiological mortality surveillance of COVID-19 in Belgium. This database is managed by Sciensano, the Belgian Public Health Institute. Sciensano adopted an inclusive definition for COVID-19 death notifications, by also including possible COVID-19 related deaths. This resulted in a robust correlation between COVID-19 and all-cause mortality. The database includes all COVID-related deaths during the first two calendar years of the pandemic (2020-2021). However, information on age and sex was missing for 35 cases and 26 cases respectively. For sex, the imputation of missing data was achieved by sampling from the marginal probabilities in the entire COVID-19 mortality dataset (i.e. $P(sex)$). For age, imputation was achieved by sampling from the distribution of age conditional on sex, and the region of death (i.e. $P\left( age \right|sex,region$)). **Table 1** contains information on the distribution of sex and age compared between the raw and imputed datasets.

**Table 1**: comparison of the distribution of sex and age by COVID-19 mortality dataset.

|  | **Raw mortality dataset** | **Imputed mortality dataset** |
| --- | --- | --- |
| Sex |  |  |
| Female  Male  Missing | 14,832 (48.0%)  16,080 (52.0%)  35 | 14,845 (48.0%)  16,093 (52.0%) |
| Age |  |  |
| Mean  SD  Missing | 81.6  11.1  26 | 81.6  11.1 |

***Abbreviations****: SD, Standard deviation*

**Appendix 3**: Comparison of YLL estimates of the COVID-19 burden in Belgium using the Global Burden of Disease Remaining Life Expectancy Table (2019) and the Belgian National Life Expectancy Table (2019).

|  | **2020** |  | **2021** |  |
| --- | --- | --- | --- | --- |
| **Absolute** |  |  |  |  |
|  | GBD 2019 RLE | BE 2019 RLE | GBD 2019 RLE | BE 2019 RLE |
| <20 years | 523 | 475 | 161 | 147 |
| 20-39 years | 3,205 | 2,816 | 2,896 | 2,547 |
| 40-59 years | 25,111 | 20,825 | 21,009 | 17,460 |
| 60-79 years | 105,183 | 80,043 | 66,813 | 51,094 |
| 80+ years | 115,662 | 71,814 | 40,092 | 25,340 |
| ***Total*** | ***249,683*** | ***175,974*** | ***130,973*** | ***96,588*** |
| **Rate per 100,000** |  |  |  |  |
| <20 years | 40.3 | 36.7 | 12.6 | 11.5 |
| 20-39 years | 217.5 | 191.2 | 196.2 | 172.6 |
| 40-59 years | 1,601.3 | 1,328.5 | 1,345.3 | 1,118.5 |
| 60-79 years | 10,019.4 | 7,588.1 | 6,088.2 | 4,633.4 |
| 80+ years | 40,279.0 | 23,246.9 | 12,673.4 | 7,488.9 |
| ***Total*** | ***2,172.5*** | ***1,531.2*** | ***1,136.8*** | ***838.3*** |
| ***Standardized^*^*** |  |  | ***1,132.4*** | ***834.6*** |

When comparing the estimated YLL for the observed burden of COVID-19 in Belgium based on the GBD 2019 reference table and the Belgian national reference table, a substantial difference can be observed. In 2020, the estimated YLL was 73,709 years larger based on the GBD 2019 table compared to the Belgian national table. In 2021, this difference was estimated at 34,385.

**Appendix 4**: Overview of distributions of disability weights approximated by a beta distribution using the 95% uncertainty interval around the DW.

The disability weights and their uncertainty intervals as proposed by the European Burden of Disease Network were considered in this study to calculate the disease burden attributable to COVID-19. Distributions for each of the disability weights that are associated with each health state were approximated by a Beta distribution from the “*prevalence*” package in R. **Figure 1** depicts the resulting approximations of these distributions for each of the included health states. **Table 1** contains the alpha- and beta-parameters of the approximated Beta-distribution for each health state.

**Figure 1**: approximation of the disability weights distribution for each health state by a beta distribution.


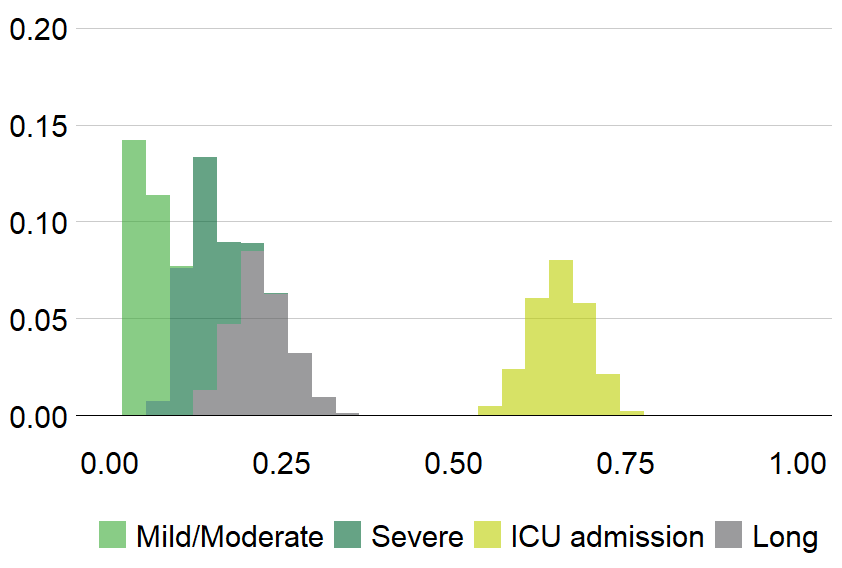


| **Table 1:** Fitted parameters based on Beta-approximation for different health states | | | | | |
| --- | --- | --- | --- | --- | --- |
| **Health state** | **Alpha** | **Beta** | **DW** | **DW LL** | **DW UL** |
| *Mild/Moderate* | 21.3 | 396.2 | 0.051 | 0.032 | 0.074 |
| *Severe* | 22.1 | 144.0 | 0.133 | 0.088 | 0.190 |
| *ICU admission* | 102.8 | 54.2 | 0.655 | 0.579 | 0.727 |
| *Long (Post-acute)* | 21.8 | 77.8 | 0.219 | 0.148 | 0.308 |
| ***Abbreviations:*** *ICU: Intensive Care Unit, DW: Disability Weight, LL: lower level, UL: upper level.* | | | | | |

**Appendix 5A**: Figure with an overview of prevalent cases and deaths in each health state for the year 2020 by day and age category.


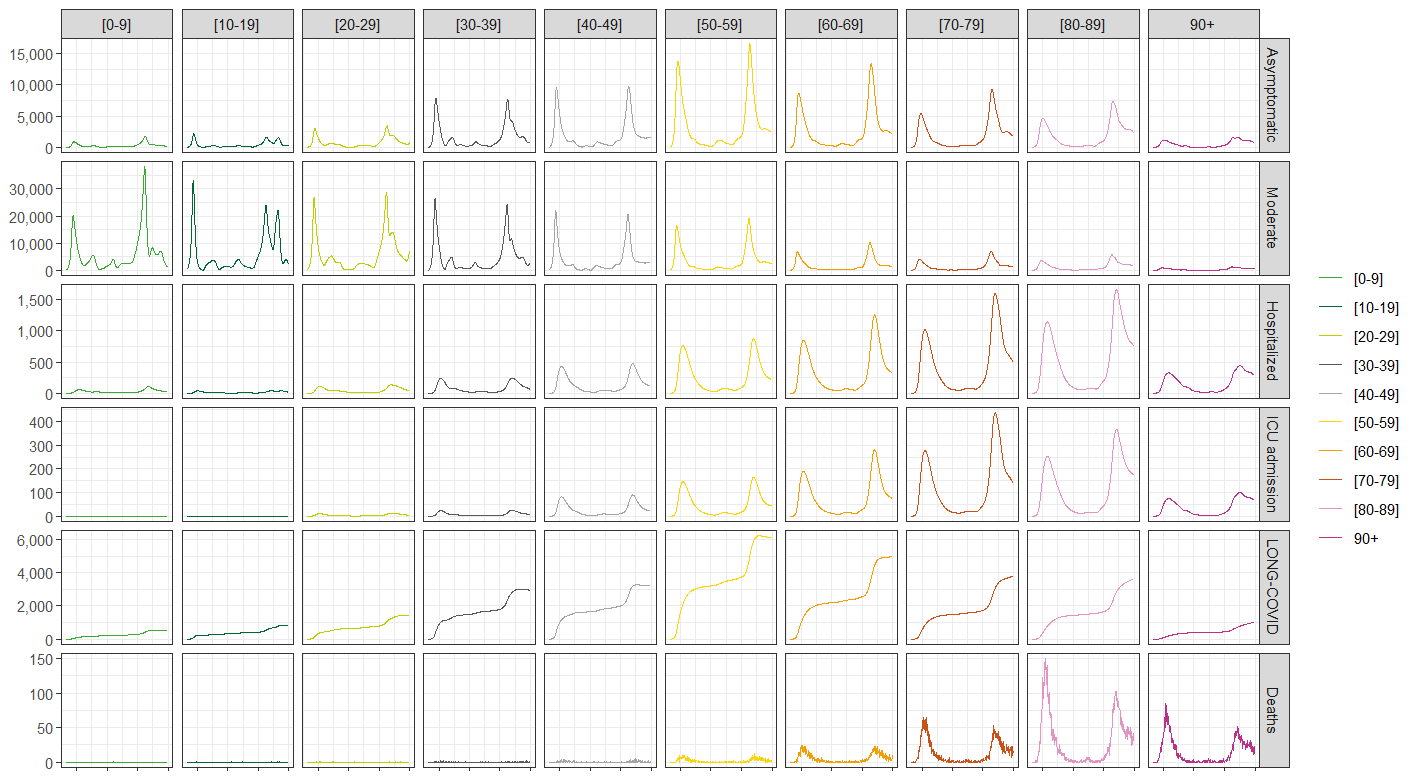


**Appendix 5B**: Figure with an overview of prevalent cases and deaths in each health state for the year 2021 by day and age category.


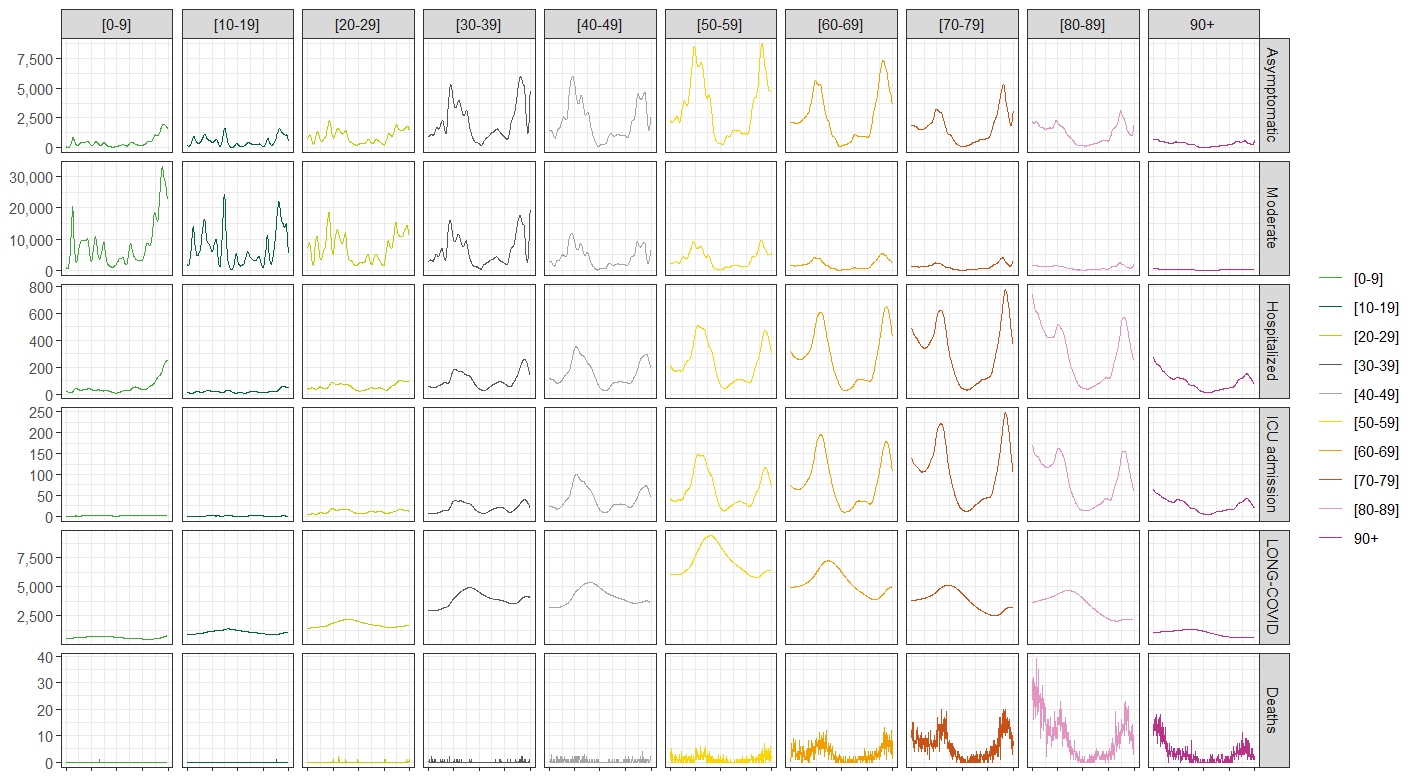


**Appendix 6**: Table with an overview of important public health measures taken in response to the COVID-19 crisis during the period 2020-2021.

| **Date** | **Public health measure** | **Details (changes)** |
| --- | --- | --- |
| 13^th^ – 18^th^ of March 2020 | Full lockdown | - Bars and restaurants are closed. - Schools are closed. - Non-essential shops are closed. - Recommendation to stay home - All social and cultural events are not permitted. |
| 4^th^ – 18^th^ of May 2020 | Slow reopening | - Close contacts are allowed among 4 people. - Opening of shops and schools |
| 3^rd^ of June 2020 | Lockdown ends | - Face masks are recommended. - Face masks are obliged when distance cannot be ensured. - Nightclubs remain closed and mass events are not permitted. |
| 25^th^ of July 2020 | Facemasks are obligated for adults | - Face masks are obliged in stores, libraries, theaters, museums… |
| 19^th^ of October – 2^nd^ of November 2020 | Gradual indroduction of partial lockdown | - Close contacts are limited to a fixed number of people. - A curfew between midnight and 5 a.m. - All non-essential stores are closed. - Restaurants and bars are closed - Schools remain closed for an additional week after the autumn break. |
| 1^st^ of December 2020 | Reopening of shops | - Non essential shops are reopened under strict conditions. |
| 5^th^ of February 2021 – 11^th^ of May 2021 | Gradual reduction of lockdown measures | Gradual reduction of remaining lockdown measures |
| 1^st^ – 15^th^ of March 2021 | Roll out vaccination | Start of vaccination campaign with priority groups including elderly, healthcare workers, and vulnerable people (e.g., people with comorbidities). |
| 1^st^ of June 2021 | Vaccination rolled out for the entire population (age ≥ 12 years) |  |
| 13^th^ of August 2021 | Introduction of Covid Safe Ticket (CST) | - People with a CST can have access to organized events. |
| 17^th^ of September 2021 | Reopening of dance and nightclubs |  |
| 20^th^ of November 2021 | Face masks are obligated inside for minors and adults (≥ 10 years) |  |
| 27^th^ of November – 29^th^ of December 2021 | Partial lockdown | - Social events are only permitted when organized by a legitimate organisation and adhere to strict constrains. - Dance and nightclubs are closed. - Face masks are obligated for minors ≥ 6 years. |
